# Demetallation of organometallic and metal-mediated reactions

**DOI:** 10.1016/j.xinn.2022.100262

**Published:** 2022-05-18

**Authors:** Chao-Jun Li

**Affiliations:** 1 Department of Chemistry and FQRNT Centre for Green Chemistry and Catalysis, McGill University, 801 Sherbrooke Street West, Montreal, QC H3A0B8, Canada

**Keywords:** organometallic reactions, C–C bond formations, C–H functionalization, sustainable chemical synthesis, umpolung hydrazones

## Abstract

The use of stoichiometric organometallic reagents and stoichiometric metals formed the basis of vast majority of classical reactions for constructing carbon–carbon bonds. The indispensable requirement of stoichiometric metals for such reactions constitutes significant challenges in terms of resource sustainability, operational safety, and chemical-waste management. The recent developments in C–H functionalizations, hydrogenative alkene/alkyne addition to electrophiles, the hydrazone umpolung chemistry, and other emerging fields such as the electrosynthesis and photoredox chemistry provide potential solutions to overcome these inherent challenges.

While Mother Nature has primarily used enzyme-catalyzed aldol reactions for generating C–C bonds for billions of years, the utilizations of stoichiometric organometallic reagents and/or stoichiometric metals for cross-couplings have formed the majority of C–C bond-formation reactions in the classical and modern chemical syntheses (Figure 1Aa and 1Ab), which are represented by the more than 30 variously named reactions in organic chemistry, such as the Grignard-type reactions, the conjugate additions of copper reagents, and the modern transition-metal-catalyzed cross-coupling reactions. Their importance in chemical synthesis is further exemplified by being the subjects of the 1912, 1979, and 2010 Nobel Prizes in chemistry.^1^ In spite of the enormous successes with these reactions and their extensive roles in synthesizing various critical modern chemical products (pharmaceuticals, agrochemicals, fine chemicals, and organic materials), looking into the future, there are significant challenges associated with the sustainability of chemical syntheses based on these celebrated reactions: (1) most organometallic reagents use organic halides as feedstocks, which are not naturally available and need to be pre-synthesized; (2) stoichiometric metals have to be mined and processed, which causes sustainability issues in the mining and metallurgy industry; (3) many of these reactions cannot tolerate reactive functional groups such as hydroxyl, amine, and carboxylic acids commonly associated with naturally abundant renewable biomasses, which require extensive functional-group protections/deprotections; (4) stoichiometric metal and/or metal halide wastes need to be dealt with; and (5) it is both highly expensive and technically problematic for large-scale applications for these reactions. The recent development of green chemistry^2^ has led to the reckoning of potential innovations of finding alternatives to the classical organometallic reactions and metal-mediated C–C bond formations without resulting to stoichiometric organometallic reagents and/or stoichiometric metals. Some promising advances have been made in the use of C–H bond as an organometallic reagent equivalent through C–H functionalization, via the use of hydrogenative addition reactions of alkenes and alkynes with electrophiles, through the umpolung of oxygenated compounds as organometallic reagent surrogates as well as the employment of electrons derived from electrochemistry and photochemistry instead of stoichiometric-metal-based reductants for certain traditionally metal-based reactions.

**Figure 1:**
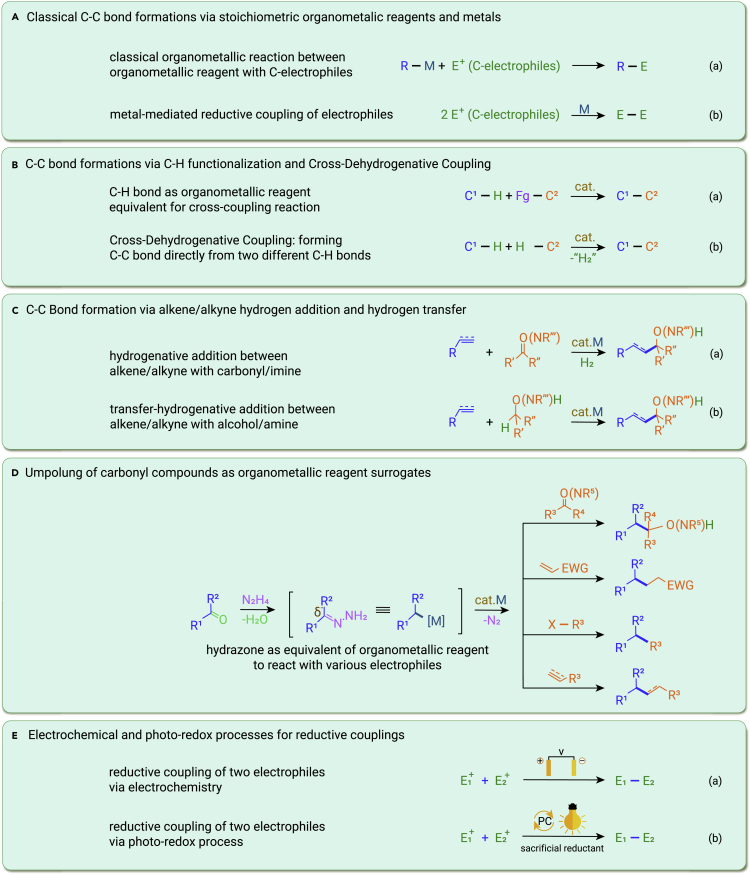
Classical organometallic reactions and organometallic-type reactions without using stoichiometric metals

## C–H functionalization

The utilization of C–H bond for the direct generation of C–C bond has a long history with prominent examples including the Friedel-Crafts reaction and the Heck-reaction for the overall functionalization of sp^2^ C–H bonds; the Glaser coupling, the Sonogashira coupling, and the more recent Aldehyde-Alkyne-Amine (A^3^) reaction for the functionalization of sp C–H bonds; and Shilov’s report in 1969 on platinum-catalyzed methane C–H activation initiated the functionalization of sp^3^ C–H bonds, with Murai’s report of catalytic-directed aryl C–H functionalization providing a turning point for the rapid development of the field, particularly toward the functionalization of sp^3^ C–H bonds recently (Figure 1Ba).^3^ Such reactions overcome the necessity of stoichiometric metals in forming the corresponding C–C bonds by the classical methods. The recent development of cross-dehydrogenative couplings, also coined as Li's cross-dehydrogenative coupling reaction, directly from two different C–H bonds by the formal removal of two H atoms has overcome the requirement of functional groups in C–C bond formations and further exemplifies the power of C–H functionalizations in chemical syntheses (Figure 1Bb).^4^

## Hydrogenative nucleophilic addition reactions with alkenes/alkynes and H_2_

Inspired by the classical catalytic-hydrogenation process, Krische has developed Ir- and Ru-catalyzed carbonyl reductive C–C bond formations by employing unsaturated hydrocarbons (e.g., alkenes, alkynes, allyl acetates, and conjugated carbonyl derivatives) together with H_2_ to circumvent issues related to stoichiometry of metals and organic halides for nucleophilic additions with organometallic reagents (Figure 1Ca).^5^ In 2005, Tu et al. reported the first coupling of alcohols with olefins.^6^ Krische further developed such reactions via the hydrogen-transfer strategy, which also allows the direct and asymmetric synthesis of chiral alcohols and amines via C–C bond formation at the α-position (Figure 1Cb). On the other hand, Buchwald developed the hydrocupration with relatively unactivated and electronically unpolarized olefins, producing alkylcopper intermediates that led to the addition of olefin-derived nucleophiles to carbonyl derivatives directly,^7^ while Montgomery developed the Ni-catalyzed reductive coupling of alkynes with carbonyls, albeit with hydrosilanes as the hydrogen source.

## Hydrazones as surrogates of orgnometallic reagents

The classical Wolff-Kishner-Huang Minlon reduction converts carbonyls to methylene derivatives mediated by hydrazine with the extrusion of N_2_ gas. The reaction mechanism involves the *in situ* generation of a carbanion intermediate. On the other hand, the vast naturally abundant biomass provides readily available oxygenated functional groups and an ideal sustainable chemical feedstock. While searching for a sustainable strategy to directly utilize these “natural functional groups” and to avoid stoichiometric metals possibly by enlisting a nitrogen cycle, Li conceptualized using umpolung hydrazones, readily generated from naturally abundant alcohols and aldehydes, as organometallic reagent surrogates for a wide range of classical organometallic reactions without using the classical stoichiometric organometallic reagents (Figure 1D).^8^ These reactions include the Grignard-type 1,2-nucleophilic additions with aldehydes, ketones, imines, and CO_2_; the homo- and cross- McMurry-type olefination; the Michael addition with various electron-deficient conjugated C=C compounds; and the Suzuki/Negishi, the Tsuji-Trost, the Ullmann, and the Heck-type cross-couplings. Furthermore, such organometallic reagent surrogates can also go beyond the classical organometallic reactions by the selective hydroalkylation of alkene, alkyne, and dienes. The reactions can also be carried out in water and for the direct functionalization of native carbohydrates.

## Other methods: Electrochemistry and photo-redox processes

When looking at the role of stoichiometric metals in the classical organometallic reagents and metal-mediated reactions, their key function is to provide electrons to turn partial positively charged carbon electrophiles into neutral or partial negatively charged species for generating C–C bonds. Electrochemistry provides a similar function by providing electrons directly without sacrificing stoichiometric metals. Although the idea of using electric current rather than metals to provide electrons for C–C bond formations goes back to the early stages of chemistry as in the Kolbe reaction, the recent focus in green chemistry has led to a rapid development of this field. Electrochemistry injects electrons directly into electrophiles on the surface of electrodes, which allows further reaction to generate C–C bonds (Figure 1Ea).^9^ Another method that can directly provide electrons to electrophiles without resorting to stoichiometric metals is via the photo-redox catalysis under photo-irradiation in the presence of a sacrificial reductant, which forms C–C bond upon subsequent reactions (Figure 1Eb).^10^ The last decade has seen an enormous development on this subject, which has become a major designing tool in chemical syntheses. Some of the classical metal-based reactions can be replaced by the electro-/photochemical processes.

## Conclusion

The utilization of stoichiometric organometallic reagents and stoichiometric metals for C–C bond formations has been the principal foundation of classical chemical syntheses. However, looking into the future, such reactions pose significant sustainability challenges. Innovations such as the recent developments in C–H functionalization, hydrogenative alkene/alkyne-H_2_ nucleophilic addition reactions, and the hydrazone umpolung chemistry as well as other emerging technologies such as the electro- and photochemical processes provide potential solutions to overcome the reliance of stoichiometric metals for chemical syntheses. Such organometallic reaction equivalents, but without resorting to stoichiometric metals, will play a major role in future chemical syntheses.
